# Nrf2 Deficiency Accelerates IL-17-Dependent Neutrophilic Airway Inflammation in Asthmatic Mice

**DOI:** 10.3390/antiox13070818

**Published:** 2024-07-08

**Authors:** Kenya Kuramoto, Yuko Morishima, Kazufumi Yoshida, Satoshi Ano, Kai Kawashima, Yuki Yabuuchi, Chio Sakai, Sosuke Matsumura, Kengo Nishino, Kai Yazaki, Masashi Matsuyama, Takumi Kiwamoto, Yukio Ishii, Nobuyuki Hizawa

**Affiliations:** Department of Pulmonary Medicine, Institute of Medicine, University of Tsukuba, Tsukuba 305-8575, Japanchio.sakai@gmail.com (C.S.); kengonishino3@gmail.com (K.N.); ishii-y@md.tsukuba.ac.jp (Y.I.);

**Keywords:** asthma, IL-17, neutrophilic inflammation, Nrf2, RORγt, Th17

## Abstract

Asthma is a heterogeneous disease that can be broadly classified into type 2, which is primarily steroid-sensitive and eosinophilic, and non-type 2, which is primarily steroid-resistant and neutrophilic. While the mechanisms leading to the development of molecular-targeted therapies for type 2 asthma are being elucidated, much remains to be learned about non-type 2 asthma. To investigate the role of oxidative stress in refractory allergic airway inflammation, we compared asthma models generated by immunizing wild-type and nuclear factor erythroid-2-related factor 2 (Nrf2)-deficient mice with the house dust mite antigen. Both asthma models had similar levels of airway inflammation and hyperresponsiveness, but the Nrf2-deficient mice had increased oxidative stress and exacerbated neutrophilic airway inflammation compared with the wild-type mice. Type 2 cytokines and the expression of GATA3, a transcription factor that is important for Th2 cell differentiation, had decreased in Nrf2-deficient mice compared with the wild-type mice, whereas helper T (Th) 17 cytokines and the expression of RORγt, which is important for Th17 cell differentiation, had increased. Furthermore, the neutrophilic airway inflammation caused by Nrf2 deficiency was ameliorated by interleukin (IL)-17 neutralization. We have concluded that the disruption of the Nrf2-mediated antioxidant defense system contributed to the induction of Th17 differentiation and exacerbated allergic neutrophilic airway inflammation.

## 1. Introduction

Asthma is a chronic inflammatory airway disease associated with symptoms such as wheezing, shortness of breath, chest tightness, and cough caused by airflow limitation and/or airway hyperresponsiveness. The inflammatory process in asthma is quite heterogeneous but can be broadly categorized into type 2 and non-type 2 inflammation. Type 2 inflammation is characterized by increased eosinophilia in the peripheral blood and airways, elevated fractional concentrations of exhaled nitric oxide, high levels of immunoglobulin E, and responsiveness to steroids, whereas non-type 2 inflammation manifests with increased airway neutrophilia and resistance to steroid treatment [1,2]. The majority of patients with asthma exhibit type 2 inflammation and respond well to steroid therapy [3], and all biologics approved to date primarily target type 2 inflammation. In contrast, effective therapies for non-type 2 neutrophilic inflammation have not yet been established.

The airways are constantly exposed to the external environment, rendering them susceptible to reactive oxygen species (ROS) arising from external and endogenous sources. Respiratory system homeostasis relies on a delicate equilibrium between ROS production and the endogenous antioxidant defense system. Nuclear factor erythroid-2-related factor 2 (Nrf2) is a member of the basic leucine zipper transcription factor family and plays a major role in the cellular antioxidative response by regulating the expression of cytoprotective genes [4,5,6]. Given its constant exposure to the environment, maintaining a balance between oxidants and the Nrf2-regulated antioxidative response is extremely important in the respiratory system. When this balance is disrupted, oxidative stress results, leading to cellular damage and contributing to the lung pathology of a variety of diseases, including pulmonary emphysema [7,8], pulmonary fibrosis [9,10], acute lung injury [11], influenza-virus-induced airway inflammation [12], and mycobacterial infection [13].

The relationship between oxidative stress and asthma severity, particularly for smoking-related asthma, has long been studied. Cigarette smoke serves as a prevalent source of excess ROS, and smoking exacerbates airway symptoms [14,15], inflammation [16], and hyperresponsiveness [17,18], increasing the risk of exacerbation [19,20] and inducing steroid insensitivity [17,21,22,23] in asthma. Some studies have reported that smoking exacerbates eosinophilic airway inflammation [18,24], while numerous others have observed the exacerbation of neutrophilic inflammation [15,17,25]. The extent of neutrophil infiltration has been suggested to correlate with the decline in pulmonary function in smoking asthmatics [16]. Additionally, smoking-induced neutrophilic airway inflammation makes steroids less effective [26], because steroids do not readily induce apoptosis in neutrophils [27]. It can be speculated that the impact of oxidative stress leads to non-type 2 airway inflammation in asthma, a factor associated with asthma severity and steroid resistance. This study aimed to elucidate the molecular mechanisms underlying non-type 2 airway inflammation, with a specific focus on impaired antioxidant defense, and to identify potential mediators that can be used as therapeutic targets using an *Nrf2*^−/−^ asthma mouse model. By inducing allergic airway inflammation in *Nrf2*^−/−^ mice raised under clean and specific-pathogen-free conditions without excess external ROS exposure, the impact of a reduced antioxidant defense response on asthma pathogenesis was examined.

## 2. Materials and Methods

### 2.1. Animal Model

Wild-type BALB/c mice were purchased from Charles River Breeding Laboratories (Kanagawa, Japan), and Nrf2-deficient (*Nrf2*^−/−^) mice of the same background were generated in our laboratory, as previously described [5,11]. The mice were 8 to 12 weeks of age and were maintained in our animal facilities under specific-pathogen-free conditions. All animal experiments were approved by the institutional review board of the University of Tsukuba and carried out according to institutional guidelines.

### 2.2. Experimental Protocols

To induce allergic airway inflammation, 8–12-week-old female mice were intraperitoneally sensitized with 40 µg of house dust mite (HDM) with an aluminum hydroxide adjuvant on day 0 and subsequently challenged with 100 µg of HDM intranasally on day 10 [28]. The control mice were sensitized and challenged with the same amount of saline. Some HDM-treated Nrf2^−/−^ mice received 0.5 mg/kg anti-interleukin (IL)-17 antibody (R&D Systems, Minneapolis, MN, USA) intraperitoneally 4 h before and 48 h after the challenge. Ninety-six hours after the last exposure to saline or HDM, airway resistance was measured, and bronchoalveolar lavage fluid (BALF) and lung tissue samples were collected.

### 2.3. Measurement of Airway Hyperresponsiveness (AHR)

The mice were anesthetized with ketamine through intraperitoneal injection and connected to a mechanical ventilator (Fine-Pointe, Buxco, Wilmington, NC, USA). Aerosolized methacholine was administered at increasing concentrations (0–50 mg/mL), and the peak airway resistance was recorded at each dose.

### 2.4. Bronchoalveolar Lavage

BALF was collected by washing the entirety of the lungs five times with 1 mL of saline through a tracheal cannula. The recovery of BALF was consistently above 75% and did not significantly differ among the animals. The total cell count was determined using a hemocytometer, and a differential cell count was calculated using standard light microscopy based on staining with Diff-Quik (American Scientific Products, McGaw Park, IL, USA). The data were normalized to the volume of BALF collected.

### 2.5. Histopathology and Immunohistochemistry

The lungs were fixed with 10% neutral buffered formalin, embedded in paraffin, and sectioned at 4 μm for histopathological analysis. The sections were stained with hematoxylin and eosin (H&E) for pathological evaluation and with direct fast scarlet (DFS) for the identification of eosinophils. For immunohistochemistry, de-paraffinized sections were processed after endogenous peroxidase quenching using the universal immuno-enzyme polymer method (Histofine Simple Stain Kit; Nichirei, Tokyo, Japan). Anti-lymphocyte antigen 6 family member G (Ly6G) monoclonal antibody (E6Z1T, 1:100, Cell Signaling Technology, Danvers, MA, USA) was used for the identification of neutrophils, anti-8-hydroxy-deoxyguanosine (8-OHdG) monoclonal antibody (N45.1, 1:100, Japan Institute for the Control of Aging, Shizuoka, Japan) was used for the evaluation of oxidative DNA damage, and nonimmune mouse IgG was used as the negative control. Diaminobenzidine (DAB) was used as a chromogen for color development, and Myer’s hematoxylin was used as a counterstain.

### 2.6. Purification of Helper T (Th) Cells

The lung tissue collected from each mouse genotype was minced, incubated in RPMI 1640 containing 10% FCS and 75 U/mL type I collagenase (Sigma-Aldrich, St. Louis, MO, USA) for 45 min, and filtered through nylon mesh to remove large tissue fragments. The Th cells were then recovered with the magnetic-bead-activated cell sorting (MACS) purification system with positive selection for CD4^+^ T cells using CD4-specific MACS microbeads (Miltenyi Biotech, Auburn, CA, USA).

### 2.7. RNA Isolation and Real-Time PCR

Total RNA was extracted from whole lung tissue or purified Th cells using an RNeasy Mini Kit (Qiagen, Venlo, Netherlands), according to the manufacturer’s instructions. First-strand cDNA was synthesized from RNA using a High-Capacity cDNA Reverse Transcription Kit (Applied Biosystems, Foster City, CA, USA), and the levels of mRNA transcripts for the target genes were quantified with real-time PCR using a THUNDERBIRD^®^ STBR^®^ qPCR Mix (TOYOBO, Osaka, Japan) and a QuantStudio 5 Thermal Cycler (Thermo Fisher Scientific, Waltham, MA, USA). The primer sequences (accession numbers) used were as follows: *interferon* (*IFN*)-*γ* (NM_008337), 5′-GCGTCATTGAATCACACCTG-3′, and 5′-TGAGCTCATTGAATGCTTGG-3′; *interleukin* (*IL*)-*4* (NM_021283), 5′-GGTCTCAACCCCCAGCTAGT-3′, and 5′-GCCGATGATCTCTCTCAAGTGAT-3′; *IL-5* (NM_010558), 5′-CTCTGTTGACAAGCAATGAGACG-3′, and 5′-TCTTCAGTATGTCTAGCCCCTG-3′; *IL-6* (NM_031168), 5′-TAGTCCTTCCTACCCCAATTTCC-3′, and 5′-TTGGTCCTTAGCCACTCCTTC-3′; *IL-17* (NM_010552), 5′-GGCCCTCAGACTACCTCAAC-3′, and 5′-TCTCGACCCTGAAAGTGAAGG-3′; *macrophage inflammatory protein* (*MIP*)-*2* (NP_033166), 5′-CCAACCACCAGGCTACAGG-3′, and 5′-GCGTCACACTCAAGCTCTG-3′; *tumor necrosis factor* (*TNF*)-*α* (NM_013693), 5′-GGCAGGTCTACTTTGGAGTCATTGC-3′, and 5′-ACATTCGAGGCTCCAGTGAATTCGG-3′; *heme oxygenase 1* (*HO-1*) (NM_010442), 5′-AAGCCGAGAATGCTGAGTTCA-3′, and 5′-GCCGTGTAGATATGGTACAAGGA-3′; *NAD*(*P*)*H:quinone-oxidoreductase-1* (*NQO1*) (NM_008706), 5′-AGGATGGGAGGTACTCGAATC-3′, and 5′-AGGCGTCCTTCCTTATATGCTA-3′; *T-bet* (NM_019507), 5′-TTCCCATTCCTGTCCTTCAC-3′, and 5′-CCACATCCACAAACATCCTG-3′; *GATA-3* (NM_008091), 5′-GGAAACTCCGTCAGGGCTA-3′, and 5′-AGAGATCCGTGCAGCAGAG-3′; *RORγt* (NM_011281), 5′-TCCACTACGGGGTTATCACCT-3′, and 5′-AGTAGGCCACATTACACTGCT-3′; and *glyceraldehyde 3-phosphate dehydrogenase* (*GAPDH*) (NM_008084), 5’-TGTGTCCGTCGTGGATCTGA-3’, and 5’-CCTGCTTCACCACCTTCTTGAT-3’. The gene expression levels for each amplicon were calculated using the ΔΔCt method and normalized to the expression of the GAPDH gene.

### 2.8. Multiplex Immunology

The levels of cytokines, including IFN-γ, IL-4, IL-5, IL-6, IL-17, MIP-2, and TNF-α, were measured in BALF with bead-based multiplex assays using Luminex technology (Luminex Corporation, Austin, TX, USA), according to the manufacturer’s instructions.

### 2.9. Western Blot Analysis

The nuclear extracts were prepared from lung tissue using the Minute™ Cytoplasmic and Nuclear Extraction Kit (Invent Biotechnologies, Inc., Plymouth City, MN, USA), according to the manufacturer’s instructions. Aliquots of nuclear extracts were separated using SDS-PAGE and transferred onto polyvinylidene difluoride (PVDF) membranes. After blocking to prevent nonspecific binding, the PVDF membranes were incubated with primary anti-Nrf2 antibody (D1Z9C; Cell Signaling Technology) overnight at 4 °C, followed by incubation with secondary antibodies (IRDye^®^ 680RD donkey anti-rabbit IgG; Li-Cor Biosciences, Lincoln, NE, USA). Immunoreactive bands were visualized with an image scan using an LI-COR Odyssey Imaging System (LI-COR Biosciences). The values were normalized to lamin B1 to evaluate the expression of Nrf2.

### 2.10. Statistical Analysis

Graph Pad Prism 9.5 software was used for statistical evaluations. The differences between the two groups were assessed with Student’s *t*-test. Multiple comparisons among the experimental groups were analyzed using one-way ANOVA, followed by the Tukey–Kramer test. Airway responsiveness to methacholine was analyzed with repeated-measures ANOVA, followed by the Tukey–Kramer test. The data are shown as the mean ± SEM, and a *p*-value of < 0.05 was considered statistically significant.

## 3. Results

### 3.1. Nrf2 Deficiency Exacerbates Neutrophilic Airway Inflammation in a Mouse Model of Asthma

**Figure 1:**
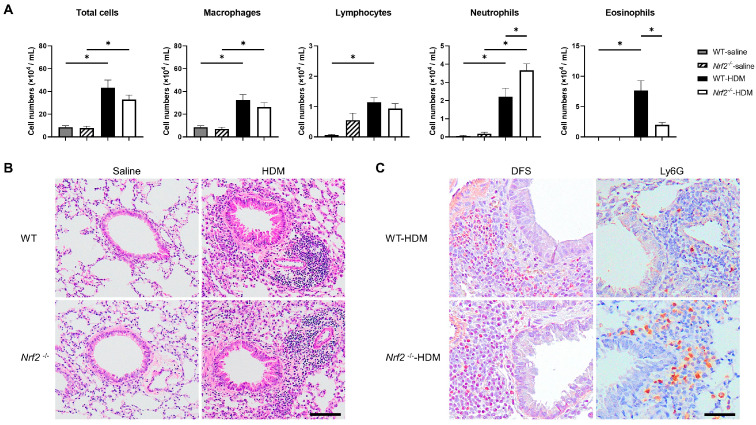
Deletion of nuclear factor erythroid-2-related factor 2 (Nrf2) exacerbated house dust mite (HDM)-induced neutrophilic airway inflammation. (**A**) Numbers of total cells, macrophages, lymphocytes, neutrophils, and eosinophils in the bronchoalveolar lavage fluid (BALF) of wild-type and Nrf2-deficient (*Nrf2*^−/−^) mice exposed to saline or HDM. The data shown are the mean ± SEM (*n* = 6–8) and are representative of three independent experiments. * *p* < 0.05, determined through one-way ANOVA, followed by the Tukey–Kramer test. (**B**) Representative images of hematoxylin and eosin (H&E) staining of lungs from wild-type and *Nrf2*^−/−^ mice exposed to saline or HDM (scale bar: 100 µm). (**C**) Representative images of staining with direct fast scarlet (DFS) and anti-lymphocyte antigen 6 family member G (Ly6G) antibody to show eosinophil and neutrophil infiltration, respectively, in the lung tissue from wild-type and *Nrf2*^−/−^ mice exposed to saline or HDM (scale bar: 50 µm). WT denotes wild-type.

The impact of Nrf2 deletion on antigen-induced allergic airway inflammation was evaluated 96 h after HDM exposure. There were no changes in the total cell numbers or inflammatory cell profiles in the wild-type or *Nrf2*^−/−^ mice in the absence of antigen exposure. However, the sensitization and exposure to HDM increased the number of neutrophils and decreased the number of eosinophils in the BALF of the *Nrf2*^−/−^ mice compared with that of the wild-type mice, although the total cell count was unchanged (Figure 1A). The pathological findings in the lung tissue were consistent with the BALF results. H&E staining showed similar levels of inflammatory cell infiltration around the bronchioles and blood vessels in both the wild-type and the *Nrf2*^−/−^ mice (Figure 1B). Immunostaining for Ly6G, a marker protein for peripheral neutrophils, and DFS staining (cytoplasmic granules of eosinophils) showed more prominent neutrophil infiltration and less prominent eosinophil infiltration, respectively, in the *Nrf2*^−/−^ mice than in the wild-type mice (Figure 1C).

**Figure 2:**
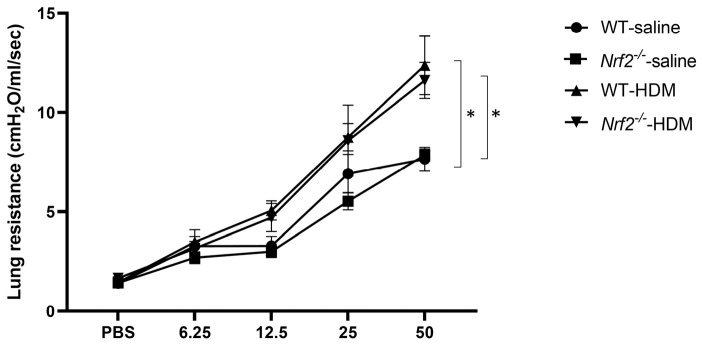
Airway hyperresponsiveness (AHR) was observed in mice with or without Nrf2 deficiency exposed to HDM. Shown here are the peak airway resistance values measured in response to increasing concentrations of methacholine (0–50 mg/mL). The data are presented as the mean ± SEM of six mice in each group and represent two independent experiments. * *p* < 0.05, determined through repeated-measures ANOVA, followed by the Tukey–Kramer test.

AHR, a physiological finding characteristic of asthma, was also assessed by measuring the resistance of the airways to aerosolized methacholine. Similar levels of increased AHR were seen in the wild-type and *Nrf2*^−/−^ mice after sensitization and exposure to HDM (Figure 2). These results suggest that the *Nrf2*^−/−^ mice exhibited an asthma-like pathology similar to that of the wild-type mice, but their inflammation was neutrophil-dominated non-type 2 inflammation rather than eosinophil-dominated type 2 inflammation.

### 3.2. Oxidative Stress Is Exacerbated in an Nrf2-Deficient Mouse Model of Asthma

**Figure 3:**
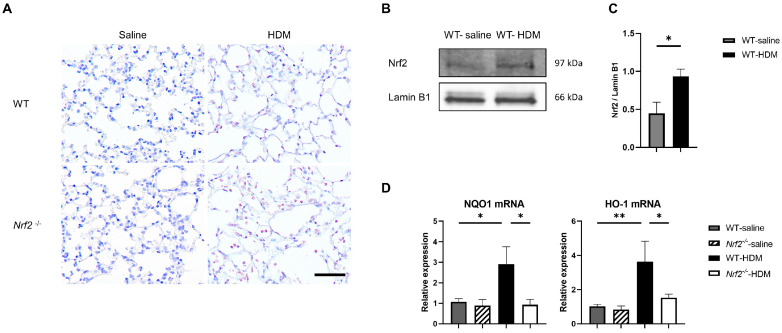
The absence of antioxidant stress response induction in *Nrf2*^−/−^ mice upon exposure to HDM resulted in increased oxidative stress in the lung tissue. (**A**) Representative immunostaining of 8-hydroxy-deoxyguanosine (8-OHdG) in lung tissue from wild-type and *Nrf2*^−/−^ mice exposed to saline or HDM (scale bar: 50 µm). (**B**) Representative Western blot of Nrf2 expression in lung nuclear extracts from wild-type mice exposed to saline or HDM. (**C**) Semi-quantitative analysis of Western blots. Values were normalized to lamin B1 and are presented as the mean ± SEM (*n* = 4). The data are representative of two independent experiments. * *p* < 0.05, determined through Student’s *t*-test. (**D**) Levels of mRNA transcripts encoding NAD(P)H:quinone-oxidoreductase-1 (NQO1) and heme oxygenase-1 (HO-1) in the lungs of each group of mice. The relative levels of gene expression were determined using the ΔΔCt method with the glyceraldehyde 3-phosphate dehydrogenase (GAPDH) gene as a reference. The data are presented as the mean ± SEM (*n* = 6–8) and are representative of two independent experiments. * *p* < 0.05 and ** *p* < 0.01, determined through one-way ANOVA, followed by the Tukey–Kramer test.

We investigated the balance between oxidative stress and antioxidant stress responses in a mouse model of asthma. Immunostaining showed that sensitization and exposure to HDM increased the expression of 8-OHdG, a marker of oxidative stress, in *Nrf2*^−/−^ mice compared with wild-type mice (Figure 3A). In wild-type mice, the expression of the transcription factor Nrf2 in the lung tissue was significantly increased, due to HDM stimulation (Figure 3B,C), as was the mRNA expression of NQO1 and HO-1, which are antioxidant genes downstream from Nrf2 (Figure 3D). In contrast, no induction of NQO1 or HO-1 was observed in the HDM-stimulated *Nrf2*^−/−^ mice, which may explain the increased oxidative stress seen in asthma, as the defense mechanism against oxidative stress was disrupted.

### 3.3. Nrf2 Deletion Alters the Expression of Inflammatory Cytokines and Chemokines

**Figure 4:**
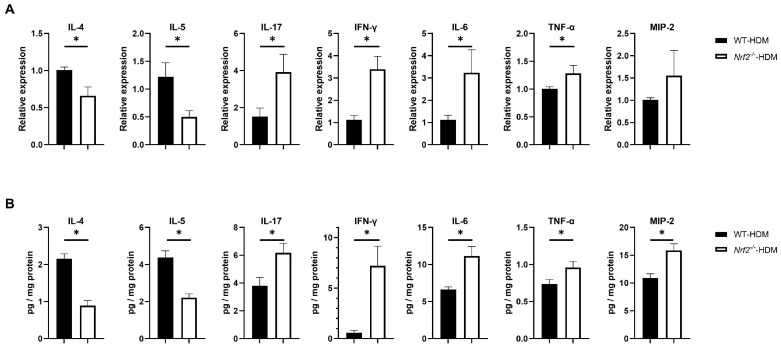
Th1, Th17, and inflammatory cytokines were overexpressed in the HDM-exposed *Nrf2*^−/−^ mice compared with the corresponding wild-type mice. (**A**) Levels of mRNA transcripts encoding IL-4, IL-5, IL-17, IFN-γ, IL-6, TNF-α, and MIP2 in the lungs of HDM-exposed wild-type and *Nrf2*^−/−^ mice. Relative quantitation of these gene transcripts was performed using the ΔΔCt method with the GAPDH gene as a reference. The data are expressed as the mean ± SEM (*n* = 4–6) and represent two independent experiments. * *p* < 0.05, determined through Student’s *t*-test. (**B**) Concentrations of IL-4, IL-5, IL-17, IFN-γ, IL-6, TNF-α, and MIP2 in lung homogenates obtained from HDM-exposed wild-type and *Nrf2*^−/−^ mice. Values were normalized to the total protein content of each sample. The data are presented as the mean ± SEM (*n* = 6–8) and represent two independent experiments. * *p* < 0.05, determined with Student’s *t*-test.

To understand how Nrf2 deficiency affects the profile of cytokines and chemokines in allergic airway inflammation, we examined the mRNA and protein expression levels of IL-4 and IL-5 (key mediators in type 2 inflammation), as well as IL-17, IFN-γ, IL-6, TNF-α, and MIP-2 (non-type 2 inflammation), in the lungs of wild-type and *Nrf2*^−/−^ mice following HDM sensitization and challenge. Compared with the wild-type mice, the levels of the Th2 cytokines IL-4 and IL-5 were significantly decreased in the *Nrf2*^−/−^ mice, while those of IL-17, IFN-γ, IL-6, and TNF-α were significantly increased (Figure 4A,B). For MIP-2, although its mRNA expression was slightly (but not significantly) increased, its protein level was significantly increased in the *Nrf2*^−/−^ mice compared with the wild-type mice (Figure 4A,B). These results suggest that Th1, Th17, and proinflammatory cytokines that induce non-type 2 inflammation tend to predominate in asthma with a reduced antioxidant stress response, while Th2 cytokines that induce type 2 inflammation are suppressed.

### 3.4. The RORγt Gene Is Overexpressed in Lung Th Cells in HDM-Stimulated Nrf2^−/−^ Mice

**Figure 5:**
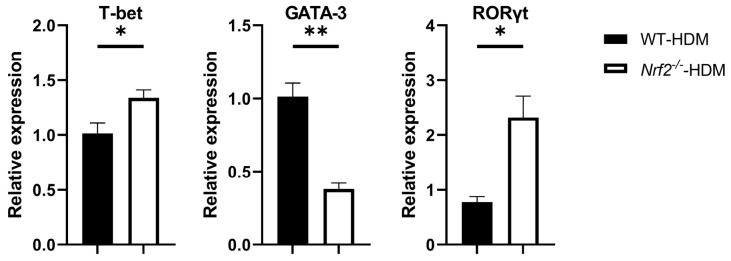
*Nrf2*^−/−^ mice exposed to HDM had a higher expression of RORγt, a key transcription factor for Th17 differentiation, than the corresponding wild-type mice did. The levels of mRNA transcripts encoding T-bet, GATA-3, and RORγt in CD4^+^ T cells recovered from the lungs of HDM-exposed wild-type and *Nrf2*^−/−^ mice. Relative quantitation of these gene transcripts was performed using the ΔΔCt method with the GAPDH gene as a reference. The data are expressed as the mean ± SEM (*n* = 3–4) and represent two independent experiments. * *p* < 0.05 and ** *p* < 0.01, determined through Student’s *t*-test.

Based on the above changes in the cytokine milieu caused by Nrf2 deficiency, we next evaluated the gene expression levels of T-bet, GATA-3, and RORγt, which are master transcription factors for the differentiation of Th1, Th2, and Th17 cells, respectively, within the lung Th cells of each group of HDM-stimulated mice. The mRNA expression of GATA-3 decreased in the *Nrf2*^−/−^ mice compared with the wild-type mice, while that of T-bet and RORγt increased, which is consistent with the changes in cytokine levels (Figure 5).

### 3.5. Treatment with Anti-IL17 Antibody Improves Neutrophilic Airway Inflammation in HDM-Stimulated Nrf2^−/−^ Mice

**Figure 6:**
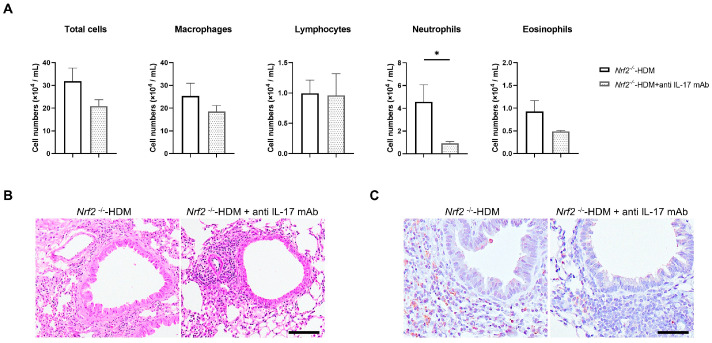
Neutralization of IL-17 reduced HDM-induced neutrophilic inflammation in the airways of *Nrf2*^−/−^ mice. (**A**) Numbers of total cells, macrophages, lymphocytes, neutrophils, and eosinophils in the BALF of HDM-exposed *Nrf2*^−/−^ mice treated with or without anti-IL-17 antibody. The data shown are the mean ± SEM (*n* = 4) and are representative of three independent experiments. * *p* < 0.05, determined through one-way ANOVA, followed by the Tukey–Kramer test. Representative images of staining with H&E (**B**) and anti-Ly6G antibody (**C**) to show neutrophil infiltration in the lung tissue from each group of mice (scale bar: 50 µm).

Since IL-17 is known to be a trigger for the induction of chemokines and proinflammatory cytokines, as well as the recruitment of neutrophils, we evaluated whether the neutralization of IL-17 ameliorates HDM-induced neutrophilic airway inflammation in *Nrf2*^−/−^ mice. Treatment with anti-IL-17 antibody decreased the neutrophil count in the BALF of the HDM-exposed *Nrf2*^−/−^ mice, while the total cell count and other cell fractions were unchanged (Figure 6A). Similarly to the results in BALF, the neutralization of IL-17 reduced Ly6G-positive inflammatory cells (neutrophils) in the airways and around blood vessels in HDM-exposed *Nrf2*^−/−^ mice (Figure 6B).

## 4. Discussion

In this study, using a mouse model of allergic airway inflammation, we first confirmed that impaired antioxidant defenses lead to non-type 2 neutrophilic airway inflammation, even in the absence of excess external ROS exposure. This finding is also consistent with our previous demonstration that Nrf2 deficiency increases susceptibility to neutrophilic airway inflammation in mouse models of elastase- or cigarette-smoke-induced emphysema [7,8]. In contrast, in a mouse model of OVA-induced asthma, type 2 airway inflammation was reported to be enhanced by cigarette smoke exposure or impaired antioxidant defense response [23,29]. Given the variability in studies regarding the nature of airway inflammation in smoking-related asthma, with some indicating increased eosinophilic inflammation [18,24] and others indicating the induction of neutrophilic inflammation [15,17,25], it can be speculated that the modification of airway inflammation through oxidative stress may be influenced by factors such as the type of external stimulus or the degree of imbalance between ROS and antioxidant defense mechanisms. Unlike OVA, HDM more closely resembles the human asthma antigen, possesses protease activity, and elicits not only an acquired immune response through major histocompatibility complex (MHC) class II, but also an innate immune response through Toll-like receptor 4 (TLR4) [30]. Another ligand for TLR4, lipopolysaccharide (LPS), has also been shown to induce neutrophilic airway inflammation in OVA-induced asthmatic mice [31]. In fact, it has been reported that a variety of asthma models can be generated using HDM rather than OVA, including type 2, non-type 2, and mixed airway inflammation [32]. Such properties of HDM were suspected to help it to induce neutrophilic airway inflammation in our asthma model.

Nrf2 is ubiquitously distributed throughout the body and becomes activated in response to inflammation-induced oxidative stress, upregulating the expression of downstream cytoprotective genes [6]. In our mouse model of HDM-induced airway inflammation, we observed an increase in Nrf2 expression in the lung tissue accompanied by the upregulation of HO-1 and NQO-1 expression. We also detected the expression of 8-OHdG (a marker of DNA oxidative damage) in lung tissue, particularly in the inflammatory cells. This expression was notably more pronounced in the *Nrf2*^−/−^ mice, which exhibited a reduced antioxidant defense response. Some previous studies have also shown a higher expression of oxidative stress markers in individuals with asthma [33], particularly in uncontrolled cases [34]. Antioxidant capacity is significantly lower in patients with poorly controlled asthma than in those with well-controlled asthma [35]. In humans, unlike in our mouse model, the interaction between the external environment and host defense mechanisms is more intricate; moreover, an increased oxidative stress burden arises from increased ROS and a diminished antioxidant defense response, exacerbating airway inflammation and perpetuating further ROS production in a vicious cycle. Naringenin, 2-trifluoromethyl-2′-methoxychalone, edaravone, and RTA-408 are known to activate Nrf2 and have been reported to suppress eosinophilic airway inflammation and AHR in a mouse model of OVA-induced asthma [36,37,38,39]. In addition, experiments with edaravone and RTA-408 have shown that they suppress eosinophilic and neutrophilic airway inflammation [38,39], which is in line with our findings. These results suggest that enhancing the antioxidative response may be promising for the treatment of both type 2 and non-type 2 inflammation in asthma.

It has been recognized that the participation of neutrophils, in addition to eosinophils, appears to be important in refractory airway inflammation, as they are associated with Th17 rather than type 2 cytokines [40,41,42,43]. Previous studies suggest that, in non-type 2 asthma, characterized by a Th17-cytokine-dominated cellular environment, steroids may not adequately suppress neutrophilic airway inflammation, AHR, or the production of inflammatory mediators [42,43,44]. IL-17, the major Th17 cytokine, is produced by a variety of cells other than Th17 cells, including cytotoxic T cells, γδT cells, group 3 innate lymphoid cells, natural killer cells, invariant natural killer T cells, mucosal-associated invariant T cells, B lymphocytes, and even neutrophils downstream from IL-17 [45,46]. Although the exact mechanism driving the IL-17-dominated cellular environment in asthma is not fully understood, our study observed an increased expression of the transcription factor RORγt, a master regulator of Th17 cell differentiation [47], in CD4+ T cells in the asthmatic Nrf2-deficient mice. Some reports suggest that increased IL-17 production induced by oxidative stress originates from airway epithelial cells [48] or γδT cells [39]; however, other studies have shown that exposure to ROS from cigarette smoke or diesel exhaust particles in HDM-induced asthmatic or healthy mice [49,50,51], or the administration of LPS to OVA-induced asthmatic mice [31], induces the differentiation of Th17 cells, with an increase in IL-17 production, which is consistent with our results. In addition, chlorine gas inhalation led to an increase in IL-17 production and airway neutrophil infiltration, but the IL-17 levels remained unaffected by neutrophil depletion [52]. Given that inhibiting IL-17 suppressed neutrophilic airway inflammation in the current study, oxidative stress may act as an upstream signal that triggers neutrophilic airway inflammation by enhancing IL-17 production by the Th17 cells. This is further supported by a study using a cockroach-allergen-extract-induced asthma model, where the administration of sulforaphane to enhance Nrf2-regulated antioxidative responses resulted in a decrease in the number of Th17 cells and IL-17 production in lung tissue [44].

In the present study, the expression of IL-6 and TNF-α in lung tissue was enhanced with increasing oxidative stress in asthmatic Nrf2-deficient mice. IL-6 and IL-1β are important cytokines activating the transcription factor RORγt [47], and mediators such as IL-23 and TNF-α have also been shown to be involved in Th17 cell polarization [53,54]. Consistently with our findings, a previous study documented that allergen stimulation resulted in elevated levels of IL-6 and IL-23 in dendritic cells (DCs) concurrently with the Th17 immune responses in lung tissue; furthermore, these responses were attenuated by Nrf2 activation in a cockroach allergen extract asthma model [44]. An additional in vitro study reported that the production of IL-6 and TNF-α was significantly elevated in dendritic cells lacking Nrf2 exposed to particulate matter compared with dendritic cells with intact Nrf2 [55]. These data suggest that these cytokines play an important role in inducing Th17 cells in response to increasing oxidative stress. The effects of oxidative stress on the Th1 and Th2 immune responses are not fully understood. In our experimental asthma model, the Th2 immune response was enhanced in the wild-type mice but attenuated in the Nrf2-deficient mice. The Nrf2-deficient mice exhibited a decreased expression of GATA3 and reduced levels of IL-4 and IL-5, whereas the expression of the Th1-related transcription factor T-bet and the levels of IFNγ increased. Puzzlingly, some studies have reported that Th2 immune responses are suppressed by Nrf2 activation [36,37,38,39], while others have reported that Th2 immune responses are enhanced, while Th1 immune responses are suppressed [56], similarly to the findings of the present study. T-bet and GATA-3 are opposing transcription factors [57], and, as seen in this study, Th1 and Th2 immunities respond in opposite directions. T-bet has also been reported to suppress not only Th2, but also Th17 immune responses [58], which is inconsistent with the findings in our asthma model. The mechanisms through which oxidative stress and Nrf2-mediated responses modulate the direction of Th1, Th2, and Th17 immune responses are complex and remain to be elucidated.

The present study has a limitation in that our mouse model does not fully replicate refractory asthma in humans. In contrast to our simplified experimental asthma model, there is a gradient of Nrf2 expression in humans, and long-term inhalation of multiple antigens and ROS, along with intricate immune and antioxidative responses, is thought to be involved in the pathogenesis of asthma. Further research is necessary to translate our findings in animal models to human applications. However, the strength of this study lies in the simplicity of the experimental model, which allowed us to observe that a reduced antioxidative response is one of the factors contributing to neutrophilic asthma. This means that, even though gene therapy targeting Nrf2 may be difficult at this time, natural bioactive compounds (such as curcumin, quercetin, resveratrol, epigallocatechin-3-gallate, apigenin, sulforaphane, and ursolic acid) [59] may improve asthmatic conditions by increasing Nrf2 activity. Furthermore, our findings demonstrate that Nrf2 deficiency accelerates IL-17-dependent neutrophilic inflammation in the airways of asthmatic mice. The lack of favorable results in a clinical trial of an anti-IL-17 receptor antibody in the treatment of asthma [60] may be attributed to an inability to identify clinical phenotypes with a greater influence of oxidative stress. The findings of the current study may indicate that an IL-17 blockade may also be effective in patients with impaired antioxidant defense responses.

## Data Availability

All data are contained within the article.
